# FindTargetsWEB: A User-Friendly Tool for Identification of Potential Therapeutic Targets in Metabolic Networks of Bacteria

**DOI:** 10.3389/fgene.2019.00633

**Published:** 2019-07-04

**Authors:** Thiago Castanheira Merigueti, Marcia Weber Carneiro, Ana Paula D’A. Carvalho-Assef, Floriano Paes Silva-Jr, Fabricio Alves Barbosa da Silva

**Affiliations:** ^1^Scientific Computing Program–Oswaldo Cruz Foundation (FIOCRUZ), Rio de Janeiro, Brazil; ^2^Laboratory of Experimental and Computational Biochemistry of Drugs (LaBECFar), Oswaldo Cruz Institute–Oswaldo Cruz Foundation (FIOCRUZ), Rio de Janeiro, Brazil; ^3^Research Laboratory in Hospital Infection (LAPIH), Oswaldo Cruz Institute–Oswaldo Cruz Foundation (FIOCRUZ), Rio de Janeiro, Brazil; ^4^Graduate Program in Biotechnology for Health and Investigative Medicine–Oswaldo Cruz Foundation (FIOCRUZ), Bahia, Brazil

**Keywords:** systems biology, flux balance analysis, metabolic network, COBRA analysis, Python (programming language)

## Abstract

**Background:** Healthcare-associated infections (HAIs) are a serious public health problem. They can be associated with morbidity and mortality and are responsible for the increase in patient hospitalization. Antimicrobial resistance among pathogens causing HAI has increased at alarming levels. In this paper, a robust method for analyzing genome-scale metabolic networks of bacteria is proposed in order to identify potential therapeutic targets, along with its corresponding web implementation, dubbed FindTargetsWEB. The proposed method assumes that every metabolic network presents fragile genes whose blockade will impair one or more metabolic functions, such as biomass accumulation. FindTargetsWEB automates the process of identification of such fragile genes using flux balance analysis (FBA), flux variability analysis (FVA), extended Systems Biology Markup Language (SBML) file parsing, and queries to three public repositories, i.e., KEGG, UniProt, and DrugBank. The web application was developed in Python using COBRApy and Django.

**Results:** The proposed method was demonstrated to be robust enough to process even non-curated, incomplete, or imprecise metabolic networks, in addition to integrated host-pathogen models. A list of potential therapeutic targets and their putative inhibitors was generated as a result of the analysis of *Pseudomonas aeruginosa* metabolic networks available in the literature and a curated version of the metabolic network of a multidrug-resistant *P. aeruginosa* strain belonging to a clone endemic in Brazil (*P. aeruginosa* ST277). Genome-scale metabolic networks of other gram-positive and gram-negative bacteria, such as *Staphylococcus aureus*, *Klebsiella pneumoniae*, and *Haemophilus influenzae*, were also analyzed using FindTargetsWEB. Multiple potential targets have been found using the proposed method in all metabolic networks, including some overlapping between two or more pathogens. Among the potential targets, several have been previously reported in the literature as targets for antimicrobial development, and many targets have approved drugs. Despite similarities in the metabolic network structure for closely related bacteria, we show that the method is able to selectively identify targets in pathogenic *versus* non-pathogenic organisms.

**Conclusions:** This new computational system can give insights into the identification of new candidate therapeutic targets for pathogenic bacteria and discovery of new antimicrobial drugs through genome-scale metabolic network analysis and heterogeneous data integration, even for non-curated or incomplete networks.

## Background

Healthcare-associated infections (HAIs), previously called hospital infections, are a serious public health problem and can develop either as a direct result of medical or surgical treatment or from being in contact with a healthcare setting. These infections include central line-associated bloodstream infections, catheter-associated urinary tract infections, ventilator-associated pneumonia (VAP), and surgical site infections. Among the pathogens related to HAI, the group of bacteria is the one that stands out. More than 2 million HAIs occur each year in the USA (Stone et al., 2005), with 50–60% being caused by antimicrobial resistant bacteria. In 2014, the World Health Organization (WHO) published the report “Antimicrobial resistance: global report on surveillance” (WHO, 2014) warning of the growing increase in antimicrobial resistance in the world. Antimicrobial resistance among hospital pathogens has increased at alarming levels, both in developed and developing countries. It is estimated that there will be a worldwide spread of untreatable infections both inside and outside hospitals. According to a bulletin published in 2017 by WHO (WHO, 2017), there are 12 major antibiotic-resistant bacteria that deserve attention and urgently need more research and development (R&D) of new and effective antibiotic treatments. Gram-negative bacteria are the most involved in HAI (carbapenem-resistant *Acinetobacter baumannii*, *Pseudomonas aeruginosa*, and *Enterobacteriaceae* family), and R&D on new antibiotics against these is considered to be of critical priority (WHO, 2017). In humans, *P. aeruginosa* is an opportunistic pathogen that causes severe infections in immunocompromised individuals. This pathogen is the main cause of morbi-mortality in patients with cystic fibrosis (Kerr and Snelling, 2009) and is a major cause of VAP.

Given the potential severity of multidrug-resistant bacteria and the lack of treatment options, the identification and implementation of effective strategies to prevent such infections are urgent priorities.

The integration of mathematical, statistical, and computational methods for biological data analysis to enable the discovery of new therapeutic targets for any bacteria is extremely relevant. The combination of bioinformatics, system modeling, and heterogeneous data integration can be a powerful tool for this purpose.

Several strategies have been proposed to search for drug targets from genome-scale models of bacterial metabolism. More often, essential genes are identified from single virtual knockouts where flux balance analysis (FBA) (Orth et al., 2010) is used to assess if this gene deletion is able to halt a selected function of bacterial metabolism. Usually, such function is biomass production (Rienksma et al., 2014). Other criteria can be combined to prioritize genes among candidate drug targets, such as existence of druggable pockets (Kozakov et al., 2015) or specificity to the bacteria as compared to the host proteins.

The construction of genome-scale metabolic network is a laborious endeavor. It combines automated steps with manual curation. The most used protocol, proposed by Thiele and Palsson (2010), lists a total of 94 steps. Nevertheless, the process is error-prone, and normally the resulting network may correctly predict some phenomena while disregarding others, which are less relevant to the study related to the reconstructed metabolic network.

The BioCyc database (Caspi et al., 2015) classifies pathway/genome databases (PGDB), each containing the full genome and predicted metabolic network of one organism, into three tiers. *Tier 1* corresponds to PGDBs that have received at least 1 year of manual curation and are updated continuously. *Tier 2* includes PGDBs that have received moderate (less than a year) amounts of review and are usually not updated on an ongoing basis. Finally, *Tier 3* refers to PGDBs that were created computationally and received no subsequent manual review or updating.

In this work, the same classification for genome-scale metabolic network models is adopted. The focus here is on metabolic network models that can be classified as Tier 2 and Tier 3, according to the BioCyc database classification. In this manuscript, *draft* metabolic reconstructions are considered Tier 3 models. Published curated metabolic models are classified as Tier 2, unless the model is identified in the literature as Tier 1.

Herein, a method for analyzing genome-scale metabolic networks of bacteria is proposed in order to identify potential therapeutic targets, along with its corresponding web implementation, dubbed *FindTargetsWEB*. The proposed method is computationally efficient, user-friendly, and robust to errors in reconstructed genome-scale metabolic networks, which are more frequent in Tier 3 (*draft*) metabolic networks. The web interface of the application is straightforward, and results are sent directly to an email address informed by the user. To demonstrate the flexibility of FindTargetsWEB, 10 genomic-scale metabolic networks of bacterial strains are analyzed in this paper. Nine of the 10 networks are available in the literature, all classified as Tier 2 models in this work: *P. aeruginosa* PAO1—version 2008 (Oberhardt et al., 2008), *P. aeruginosa* PAO1—version 2017 (Bartell et al., 2017), *P. aeruginosa* PA14 (Bartell et al., 2017), *Klebsiella pneumoniae* (Liao et al., 2011), *Haemophilus influenzae* (Schilling and Palsson, 2000), a host-pathogen genome-scale reconstruction based on the *Mycobacterium tuberculosis* metabolic network (Bordbar et al., 2010), *Staphylococcus aureus* (Becker and Palsson, 2005), and *Pseudomonas putida* (Puchałka et al., 2008). Results are also presented for two metabolic networks of *P. aeruginosa* CCBH4851, which is a multi-drug resistant strain belonging to a clone endemic in Brazil (*P. aeruginosa* ST277) (Silveira et al., 2014). Both reconstructions of *P. aeruginosa* CCBH4851 were made by our group. One reconstruction can be classified as Tier 3, and the other is the corresponding curated version, classified as Tier 2.

The web application proposed in this work combines FBA, flux variability analysis (FVA) (Gudmundsson and Thiele, 2010), extended Systems Biology Markup Language (SBML) parsing, and heterogeneous data integration in order to identify the most promising therapeutic targets. All SBML files processed in this work are available as Supplementary Material. The underlying hypothesis related to FVA is that reactions which the maximum flux is equal to the minimum flux (i.e., flux range equal to zero), given the optimal biomass production, are less robust to potential perturbations. Indeed, a high rigidity for a given reaction flux (i.e., flux range equal to zero) may indicate that the flux through this reaction is crucial for sustaining optimal growth, while a lower rigidity (i.e., flux range greater than zero) indicates that there might be alternate pathways to carry the reaction flux (Oberhardt et al., 2010). Flux ranges fell into three categories: i) inflexible fluxes (flux range equal to zero), ii) fluxes with bounded flexibility (flux range greater than zero, but bounded), and iii) infinitely flexible fluxes (flux range greater than zero, unbounded). The FVA analysis carried out by FindTargetsWEB aims to identify potential targets associated with inflexible fluxes, i.e., flux range equal to zero. The genome-scale metabolic network analysis is combined with several queries to multiple public repositories, such as KEGG (Ogata et al., 1999), UniProt (UniProt, 2018), and DrugBank (Wishart et al., 2008), to assess the druggability and toxicology of potential targets. FindTargetsWEB has identified potential targets for all networks. Several of the potential targets have been described in the literature. Other targets are candidates for future experimental investigation.

## Implementation

**Figure 1:**
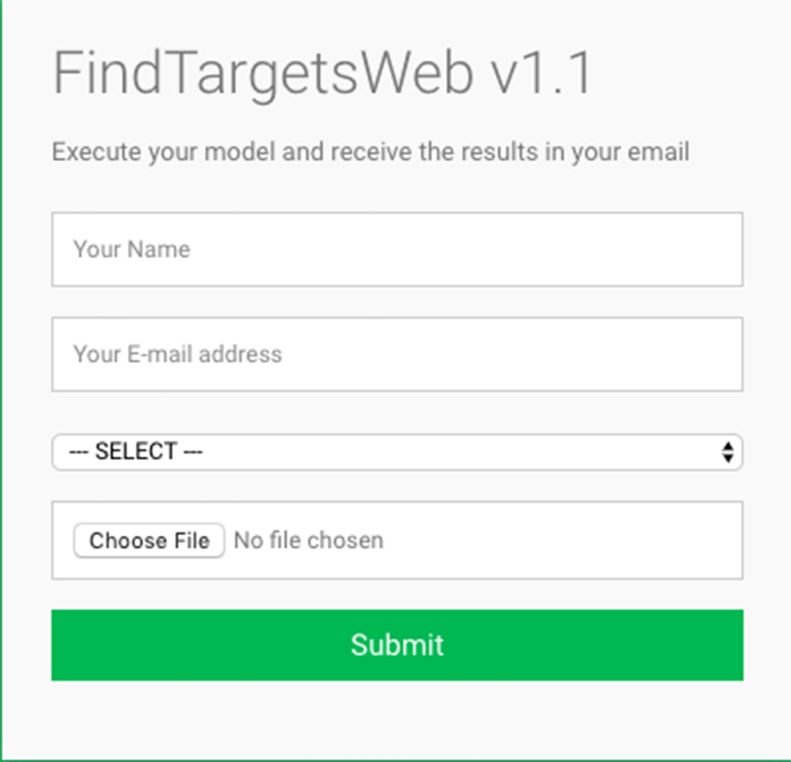
FindTargetsWEB user interface—SBML file input.

**Figure 2:**
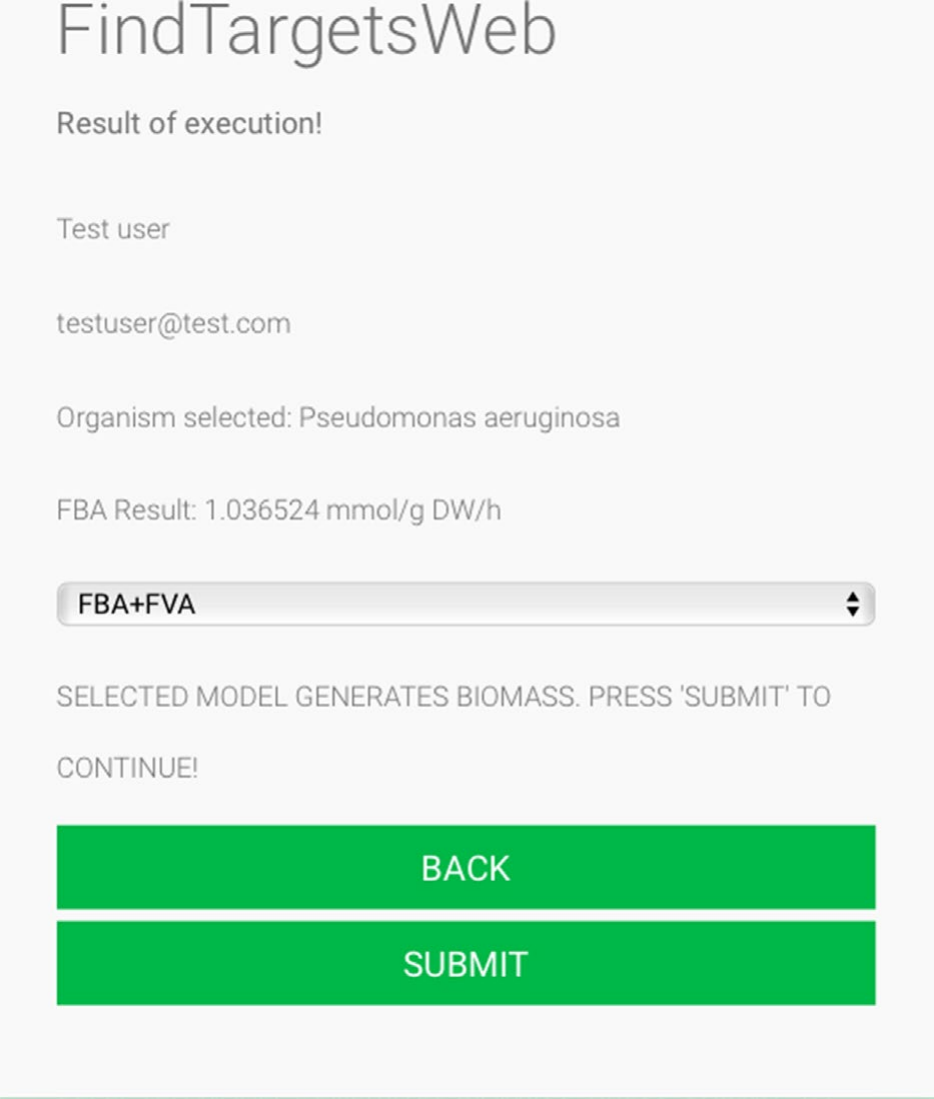
FindTargetsWEB user interface—choice of analysis method.

Some of the main requirements related to the implementation of the general method described in this work, dubbed FindTargetsWEB, were ease of use, availability, robustness, and performance. After careful consideration, Python was selected as the implementation language. Python is a high-level, interpreted, scripted, imperative, object-oriented, dynamic, and strongly typed programming language created by Van Rossum and Drake (2003). Its many advantages favor the fulfillment of the main requirements of the application. Another advantage is the availability of the COBRApy package. *COnstraint-Based Reconstruction and Analysis Toolbox* (COBRA) (Hyduke et al. 2011) methods are widely used for genome-scale modeling of metabolic networks in prokaryotes and eukaryotes. The COBRA Toolbox for MATLAB is a leading software package for analyzing metabolism on a genomic scale. On the other hand, COBRApy (Ebrahim et al., 2013) is a Python module that provides support for basic COBRA methods. COBRApy is designed in an object-oriented way, which facilitates the representation of the complex biological processes of metabolism. COBRApy does not require MATLAB to work; however, it includes an interface to the COBRA Toolbox for MATLAB to facilitate the use of legacy codes. To improve performance, COBRApy includes parallel processing support for computationally intensive processes. FindTargetsWEB is implemented as a web application. Therefore, the user only needs a web browser to access the system. The system interface is intuitive: the user needs to provide the SBML file describing the metabolic network reconstruction, the organism species associated with the metabolic network reconstruction, which defines a filter to KEGG queries, and information such as name and e-mail address (**Figure 1**). It should be emphasized that the FindTargetsWEB list of analyzable species is easily expandable and can include both gram-negative bacteria, gram-positive bacteria, and bacteria that cannot be classified as either gram-positive or gram-negative. In the following screen, the user decides if he/she wants to analyze the network using the FBA method alone or a combination of the FBA+FVA methods (**Figure 2**). The FBA+FVA method pinpoints reactions and associated genes in which knockout completely stops (zeroes) biomass generation and has an FVA range of zero. Therefore, the FBA+FVA method is more restrictive than the FBA-only option. It should be highlighted that the targets found by the FBA+FVA method compose a proper subset of the set of targets found by the FBA-only method. Robustness is provided by the design of the method itself, as described in the following paragraphs.

Target identification is carried out through a computational workflow that runs the metabolic network analysis and pinpoints genes whose virtual knockout interrupts the generation of biomass. Therefore, the minimum level of curation required for a metabolic network model to be processed by FindTargetsWEB is to have a biomass reaction flux greater than zero. The list of potential targets is filtered using FVA (if the user decides to do so), and the workflow retrieves possible inhibitors for the identified genes, verify if such inhibitors are available as approved drugs, and evaluate their toxicity to humans by querying several repositories.

**Figure 3:**
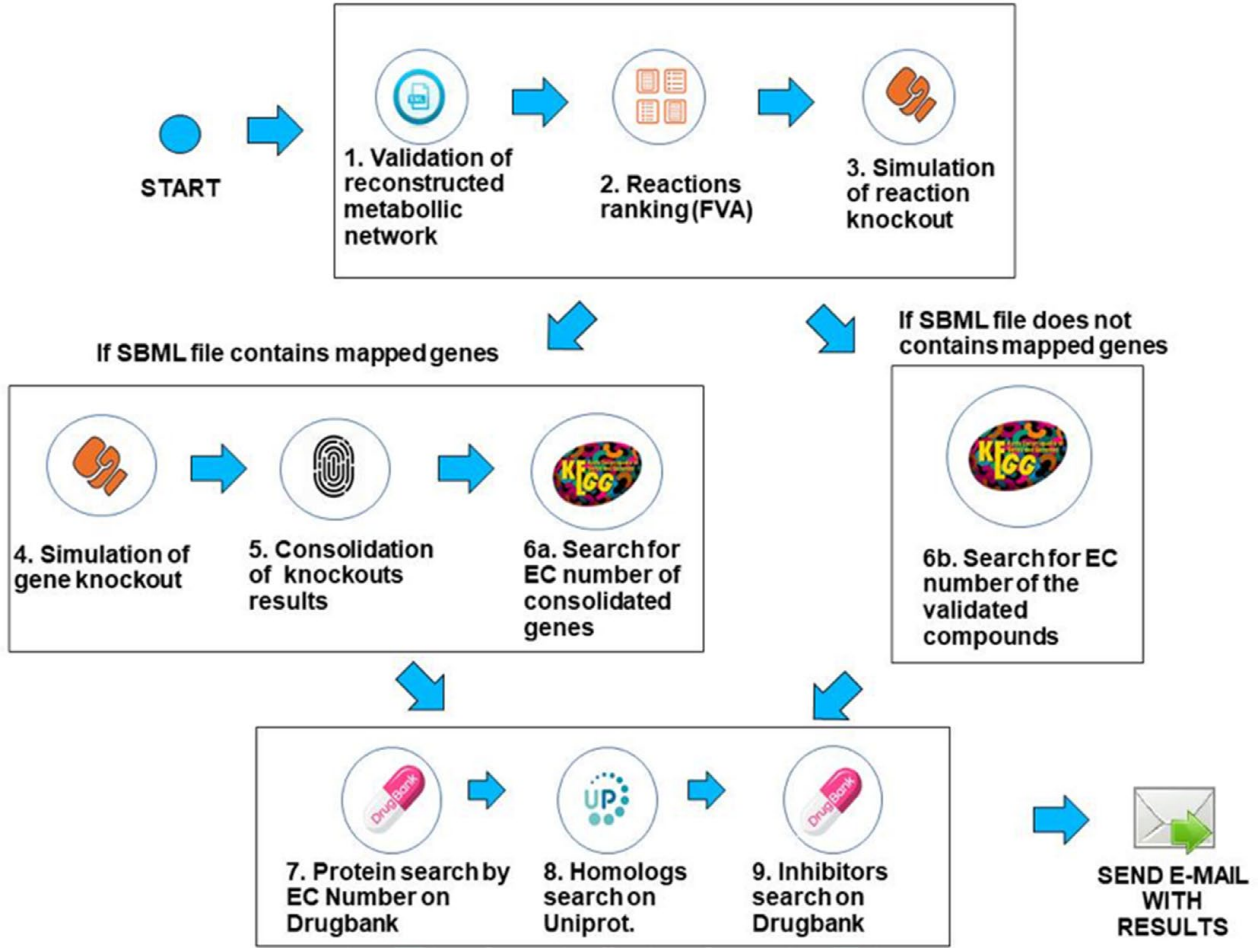
Description of the computational workflow.

The workflow was implemented using the Python programming language, version 3.6.3, and the COBRApy framework version 0.9.0. This framework has the necessary methods for reading the SBML (Hucka et al., 2015) file that describes the genome-scale metabolic network of the bacterium under analysis. The solver used for FBA and FVA analysis is GLPK (https://www.gnu.org/software/glpk/), which is the COBRApy default solver that is easily deployable on Linux platforms. The system is deployed in an Ubuntu v18.04 server with 64GB RAM. Prior to processing, when needed, SBML files were converted to the SBML level 3 format using the command cobra.io.sbml3.write_sbml_model from COBRA. The SBML files processed in this manuscript were retrieved from the BioModels repository (Glont et al., 2017) or directly from the supplementary material of the associated reference. The main steps of the method are described below. The whole method is depicted in **Figure 3**.

**1. Validation of the SBML file describing the genome-scale metabolic network**—In this step, the system first creates a table containing gene/reaction/metabolite data obtained from the SBML file and then checks if the metabolic network reconstruction generates biomass. This is done through the FBA method, considering the biomass reaction as the target for maximization. If the biomass value is zero, the system outputs an error to the user and halts processing. If the maximum flux of the biomass reaction is greater than zero, the workflow proceeds to the next step.
**2. Use of FVA to filter reactions**—After validating the metabolic network, reactions are filtered using the FVA method, if the user has decided to analyze the metabolic network using a combination of the FBA+FVA methods. The objective is to consider, in the following processing steps, those reactions which the range of possible flux values is equal to zero, given the optimal biomass generation value determined in the previous step. The underlying assumption is that reactions with a range equal to zero are less robust, i.e., more susceptible to perturbations, as stated in the introduction. Note that the FVA method can be implemented in a computationally efficient way (Gudmundsson and Thiele, 2010), and the cost of FVA analysis on the overall execution time of FindTargetsWEB is negligible.
**3. Simulation of reaction knockout**—In this step, single reaction knockouts are performed. The process is done by zeroing the maximum and minimum reaction flux constraints and running FBA again, for each reaction in the network. If biomass generation is zeroed when knocking out a given reaction, its information is stored in a list for further processing. If gene IDs are available in the SBML file, the workflow proceeds to step 4. Otherwise, it jumps directly to step 6b.
**4. Simulation of gene knockout**—In this step, the system performs single knockouts for each gene described in the model, where the COBRApy framework queries the reactions that are linked to the selected gene and zeroes the minimum and maximum value of each reaction bound to the gene, taking into account gene-protein-reaction (GPR) relations. In the same way as the previous step, if the value of the generation of biomass has zeroed, the corresponding gene information is stored in a second list. It is worth noting that one gene can be associated with more than one reaction, and one reaction may require the expression of several genes.
**5. Consolidation/unification of knockouts results**—In this step, both lists generated in the previous steps are unified, i.e., the list of reactions generated in step 3 and the gene list generated in step 4. In order to a gene to be included in the final list, it should be included in the list of step 4 and be associated with at least one reaction stored in step 3 (see Algorithm 1). These are the candidate genes that the workflow is going to consider in the following steps. It should be highlighted that the final list is filtered according to the FVA processing performed in step 2, if the option FBA+FVA is selected by the user.

**Algorithm 1**: Consolidation of knockout results (SBML with mapped genes)

**Input**: List of genes from knocked-out reactions/list of knocked-out genes

**Output**: Unified list of target genes in a text file


	1:	**procedure **UnificationTargetsList(targetGeneListFromReact, targetGeneList)
	2:			read targetGeneListFromReact
	3:			read targetGeneList
	4:			**open file** “targetgenes.txt”
	5:			**for all** targetgene **in** targetGeneListFromReact **do**
	6:				**if** targetgene **in** targetGeneList **then**
	7:				write targetgene in file “targetgenes.txt”
	8:				end if
	9:			end for
	10:			**close file** “targetgenes.txt”
	11:	end procedure

**6a. Search for EC numbers of consolidated genes.** In this step, the system queries the KEGG repository to obtain the EC number of each gene included in the final gene list obtained in the previous step (file “targetgenes.txt”). KEGG (Kyoto Encyclopedia of Genes and Genomes) is a knowledge base for systematic analysis of gene functions, linking genomic information with higher order functional information (Ogata et al., 1999). This step is important because drug retrieval in DrugBank requires the associated EC number. The result of this step is a list of EC numbers associated to their respective genes. The workflow then proceeds to step 7.
**6b. Search for EC number using reaction information.** If gene IDs are not available in the SBML file, which may be the case in draft (Tier 3) metabolic network models, EC numbers are retrieved from KEGG based on reaction information. This step is particularly important for incomplete metabolic reconstructions that do not include GPR relations and is directly related to the application’s requirement of robustness to incompleteness on metabolic network data. The KEGG search is performed using all the compounds involved in the corresponding reaction. See Algorithm 2 for a detailed description of the processing related to this step. It is worth emphasizing that this step is executed only for incomplete descriptions of genome-scale metabolic networks. The complexity of Algorithm 2 is *O(C)*, where *C* is the number of compounds included in the SBML file.

**Algorithm 2**: Search for EC numbers using reaction information (SBML without mapped genes)

**Input**: List of chemical compounds of reaction

**Output**: List of EC numbers found


	1:	procedure alternativeStepToGetECNumberWithoutGenes(listCompoundFromSBML)
	2:	# file with all compounds in SBML.
	3:	**read** listCompoundFromSBML
	4:
	5:	# Instance of biomodels python module
	6:	k <- KEGG instance
	7:
	8:	# setting timeout in seconds
	9:	k.timeout <- 200000
	10:
	11:	# All compounds in SBML file. Ex.: 2 A –> B
	12:	**for all** compound **in** listCompoundFromSBML **do**
	13:
	14:			# find all stoichiometric values with regex method in compound. Ex.: A –> B
	15:			compound_no_stoich <- remove all stoichiometric values in compound
	16:			param_splt <- empty
	17:
	18:			# Verify if reaction is reversible or irreversible
	19:			**if** “< = >“ in compound_no_stoich **then**
	20:				param_splt <- “< = >“
	21:			**else**
	22:				param_splt <- “–>“
	23:			**end if**
	24:
	25:			# Separate compounds in reactant and product. Ex.: compound_splt = [‘A’, ‘B’]
	26:			compound_splt <- compound_no_stoich.split(param_splt)
	27:
	28:			# If compound belongs to a transport reaction (influx or eflux), jump to next iteration
	29:			**if** compound_splt.length < 2 **then**
	30:				**continue**
	31:			**end if**
	32:
	33:			list_ec_number_0 <- initialize empty list
	34:			list_ec_number_1 <- initialize empty list
	35:	
	36:			# Start iterating compound_splt list with reactant and product
	37:			**for** (x = 0,1) **do**
	38:
	39:				# Get reactant or product in this variable
	40:				item_compound <- compound_splt[x] without spaces
	41:			list_id_cpd_KEGG <- initialize empty list
	42:		
	43:			# If reactant or product contains “+”, find ID in KEGG for components.
	44:			# Else, find ID in KEGG for only one component.
	45:			**if** “ + “ in item_compound **then**
	46:				item_compound_splt <- item_compound.split(“ + “)
	47:				**for all** cpd_item **in** item_compound_splt **do**
	48:					# find id compound in KEGG for cpd_item
	49:					# and insert in ids list
	50:					result_id_cpd <- k.find(“compound”, cpd_item)
	51:					insert result_id_cpd in list_id_cpd_​KEGG
	52:				**end for**
	53:			**else**
	54:				result_id_cpd <- k.find(“compound”, item_​compound)
	55:				insert item_compound in list_id_cpd_KEGG
	56:			**end if**
	57:		
	58:			# Here, all list_id_cpd_KEGG are concatenated
	59:			# found to search the reaction in KEGG.
	60:			# In Python, if list_id_cpd_KEGG length is less than 2,
	61:			# don’t put the “+” in end of string.
	62:			str_item_compound_in_cpd <- list_id_cpd_KEGG concat with “+”
	63:		
	64:			# find all reactions in KEGG with IDs of compounds
	65:			result_link_reactions_cpd <- k.link(“reaction”, str_item_​compound_in_cpd)
	66:		
	67:			# All results of result_link_reactions_cpd are inserted here
	68:			set_id_reaction_KEGG <- insert all reactions found in KEGG.
	69:		
	70:			# find all EC numbers in KEGG with reactions IDs
	71:			# in set_id_reaction_KEGG and insert in result_list_ec
	72:			result_list_ec = k.link(“enzyme”, set_id_reaction​_​KEGG)
	73:			**if** x = 0 **then**
	74:				insert result_list_ec in list_ec_number_0
	75:			**else**
	76:				insert result_list_ec in list_ec_number_1
	77:			**end if**
	78:
	79:		**end for**
	80:
	81:		list_ec_number_intersect <- initialize empty list
	82:		txt_file <- initialize txt file
	83:	
	84:		# Starts to iterate the list of ECs to identify intersections
	85:		# If found, related EC numbers are written in a text file
	86:		**for all** ec_number_0 **in** list_ec_number_0 **do**
	87:			**if** ec_number_0 in list_ec_number_1 **then**
	88:				record ec_number_0 in a txt_file
	89:			**end if**
	90:		**end for**
	91:	
	92:	**end for**
	93:
	94: **end procedure**

**7. Search for EC numbers on DrugBank**—With the EC numbers obtained in the previous steps, the system queries the DrugBank repository to verify if this database has any record of the listed EC numbers. The DrugBank database is a repository that combines detailed drug data with comprehensive drug target information (Wishart et al., 2008). If an exact match is found, the system retrieves the values of the name of the protein, organism, and UniProt ID.

When executing this query, the protein retrieved can be mapped in another organism, distinct from the target bacterium. Thus, the next step (step 8) is necessary to confirm whether the protein retrieved has a homologue in the target bacterium. Clearly, exact matches are also possible. In any case, the retrieved data is validated in the next step.

**8. Search for homologues on UniProt**—Finally, the system searches for sequence similarity between the proteins described by UniProt IDs retrieved in the last step and the proteins encoded by the genome of the target bacterium using the BLAST (basic local alignment search tool) (Altschul et al., 1990) application deployed in the UniProt server. If there is a hit (i.e., sequence similarity above 30%), all corresponding data concerning the homologue found is stored.

In this step, the homology between the target protein and human proteins is also considered. If the sequence similarity with a human protein is greater than the similarity with the target bacterium, the protein under analysis is discarded, since the inhibition of that protein could be harmful to the host. Otherwise, several data are stored, such as metabolic pathway, function, and catalytic activity, among others. This step of the workflow is the most time-consuming, since BLAST is executed for all proteins identified in the previous step.

**9. Search for existing inhibitors**—The last step is to query the DrugBank repository, using the stored UniProt IDs, in order to retrieve known inhibitors, if available. After this last step, the system generates spreadsheets containing all results that are sent to the user in a compressed file.

This method presents as results candidate genes that, when knocked-out, will cease the biomass production of the microorganism. Candidate genes must be associated with potential drug targets in DrugBank, and their sequence similarity to human proteins is also checked. The application then identifies available ligands, most often inhibitors, to the selected genes.

### System Output

Results of FindTargetsWEB’s analysis are sent to the user as a compressed file, to the e-mail address informed at the start of execution. Five spreadsheets are included in the compressed file:

- *08-filter_ECNumbers_DrugBank*—This spreadsheet contains the EC number of putative targets, along with product, organism name, UniProt ID, and DrugBank ID
- *11- hits_Uniprot*—This spreadsheet contains additional information related to UniProt queries, such as percentage of sequence similarity, BLAST e-value, gene name, pathway, function, and catalytic activity.
- *13-list_inhibitors_per_target*—This spreadsheet lists all inhibitors found for all targets. Included information are drug name, drug group (e.g. experimental, approved, investigational), and drug action.
- *14-list_inhibitors_approved*—This spreadsheet lists all inhibitors with approved drugs found for all targets. Included information are drug name, drug group (approved), and drug action.
- *model_data*—This spreadsheet lists data related to the input SBML file, such as gene IDs and associated reactions. The complete information of which reactions are associated with each gene in the metabolic network model is included in this file.
- *summary_results*—This spreadsheet contains a summary​ of data included in the previous files. Included fields are EC numbers, product, organism name, gene name, pathway, function, catalytic activity, drug name, drug group, and drug action.

## Results

In this section, analysis results for several strains of *P. aeruginosa*, *K. pneumoniae*, *H. influenzae, S. aureus, P. putida*, and a host-pathogen genome-scale reconstruction based on the *M. tuberculosis* metabolic network are presented. It should be highlighted that FindTargetsWEB can carry out analysis for other bacterial species, as indicated by the list box on the initial web page of the application. Indeed, even this list can be easily expanded to include additional species of interest, through a user request to FindTargetsWEB support team.

### Analysis of Metabolic Network Models of *P. aeruginosa*

To evaluate the accuracy of results for several metabolic networks, initially, the analysis of four metabolic networks of *P. aeruginosa* is discussed. A survey of the literature is also presented to confirm the feasibility of the candidate genes as antibacterial drug targets. Gene function and related pathways are also considered in the evaluation of results.

The four metabolic networks of *P. aeruginosa* strains analyzed by FindTargetWEB were: PAO1 version 2008—iMO1056 (BioModels ID 1507180020) (Oberhardt et al., 2008), PAO1 version 2017—iPAE1146 (Bartell et al., 2017), PA14—iPAU1129 (Bartell et al., 2017), and a curated version (Tier 2) of the metabolic network of *P. aeruginosa* CCBH4851 (Silveira et al., 2014). The SBML level 3 file describing the Tier 2 *P. aeruginosa* CCBH4851 network is available as supplementary material, as well as the SBML files of the other networks considered in this paper. It is worth noting that each metabolic network model presents a different value for the growth rate after validation of biomass generation by FBA; for PA01 version 2008, the growth rate corresponds to 1.047929 h^-1^; PA01 version 2017 has a growth rate of 15.509635 h^-1^; for the PA14 model, the growth rate is 15.508373 h^-1^, and the Tier 2 CCBH4851 model has a growth rate of 1.036524 h^-1^. Differences in growth rate among metabolic network models are due to the distinct biomass equations, as well as variation in the number of genes, reactions, and metabolites in each of the metabolic network models.

It should be mentioned that the growth rates associated with the PA14 and PAO1-2017 (Bartell et al., 2017) models depart by far from the observed growth rates of *P. aeruginosa* spp., which may vary between 0.3 and 0.8 h^-1^, depending on cultivation conditions (Brown, 1957) (Seto and Noda, 1982) (Yang et al., 2008). Nevertheless, FindTargetsWEB can still process those networks. The only requirement is to have a growth rate greater than zero.

### Description of Common Targets for *P. aeruginosa* Networks

In this subsection, common targets for all Tier 2 *P. aeruginosa* networks are listed. The metabolic network models of *P. aeruginosa* analyzed in this subsection are described at Oberhardt et al., 2008 (PAO1) and Bartell et al., 2017 (PAO1 and PA14). The *P. aeruginosa* CCBH4851 metabolic network is being modeled by our group and represents a bacterium found in a catheter of a patient hospitalized at the Brazilian state of Goiás (Silveira et al., 2014). It is worth highlighting that the Bartell et al. (2017) networks focused on modeling virulence factors. Due to this fact, the biomass equation received less attention and the growth rate is not inside the range observed for *Pseudomonas* spp. Nevertheless, the workflow was able to process both networks and found several targets common to other metabolic reconstructions. The number of unique targets found in each network, for both FAB+FVA and FBA-only methods, are listed in **Table 1**. The spreadsheet detailing all targets found is available as supplementary material.

**Table 1:** Number of unique targets found in the Tier 2 metabolic networks of *P. aeruginosa*.

	FBA-Only	FBA+FVA
PAO1-2008	53	50
PAO1-2017	50	42
PA14	44	42
CCBH4851	50	17

For the FBA-only method, 25 targets are common to all four networks. For the FBA+FVA method, 11 targets are common to all four networks.

It is important to highlight some of the genes identified as common targets for all four metabolic network models of *P. aeruginosa* (**Table 2**). The *murA* (EC 2.5.1.7) and *murB* (EC 1.3.1.98) genes encode enzymes involved in bacterial cell wall synthesis and have been identified as essential in both *Pseudomonas* spp. and *Escherichia coli* (Benson et al., 1996). The *folP* gene product (EC 2.5.1.15) is important for folic acid biosynthesis, which is fundamental for bacterial growth and reproduction (Dallas et al., 1992). The *folA* gene product (EC 1.5.1.3) is related to the biosynthesis of cofactors, being an important intermediary of folate metabolism. It is considered the key enzyme of this process and essential for microbial growth (Myllykallio et al., 2003). Another target worth mentioning is the *aroE* gene (EC 1.1.1.25), which has been described as a potential therapeutic target of both *P. putida* and *E. coli* (Peek et al., 2014).

**Table 2:** Potential targets common to all Tier 2 *P. aeruginosa* metabolic network models. Common targets identified by both FBA-only and FBA+FVA methods are marked with asterisks (*). The other targets were identified by the FBA-only method but not by the FBA+FVA method.

EC Number	Gene Name	Product	DrugBank Inhibitor
1.1.1.100	*fabG*	3-oxoacyl-[acyl-carrier-protein] reductase FabG	E
1.1.1.25	*aroE*	Shikimate dehydrogenase	E
1.17.1.8	*dapB*	4-hydroxy-tetrahydrodipicolinate reductase	E
1.3.1.98	*murB*	UDP-N-acetylenolpyruvoylglucosamine reductase	A/E
1.5.1.3	*folA*	Dihydrofolate reductase	A/E
2.1.1.45	thyA*	Thymidylate synthase	E
2.3.1.41	*fabB*	3-oxoacyl-[acyl-carrier-protein] synthase 1	A/E
2.4.1.227	murG	UDP-N-acetylglucosamine–N-acetylmuramyl-(pentapeptide) pyrophosphoryl-undecaprenol N-acetylglucosamine transferase	E
2.4.2.14	purF*	Amidophosphoribosyltransferase	E
2.5.1.15	folP*	Dihydropteroate synthase	A
2.5.1.6	metK*	S-adenosylmethionine synthase	E
2.5.1.7	*murA*	UDP-N-acetylglucosamine 1-carboxyvinyltransferase	A/E
2.6.1.16	*glmS*	Glutamine—fructose-6-phosphate aminotransferase [isomerizing]	E
2.6.1.85	*pabB*	Para-aminobenzoate synthase component 1	A
2.7.4.25	*cmk*	Cytidylate kinase	E
2.7.7.23	glmU*	Bifunctional protein GlmU	E
3.1.3.1	phoA*	Alkaline phosphatase	E
4.1.3.38	pabC*	Aminodeoxychorismate lyase	E
4.2.1.24	hemB*	Delta-aminolevulinic acid dehydratase	A/E
4.2.3.5	*aroC*	Chorismate synthase	A
5.3.1.1	*tpiA*	Triosephosphate isomerase	E
5.3.1.6	*rpiA*	Ribose-5-phosphate isomerase A	A/E
6.3.2.13	murE*	UDP-N-acetylmuramoyl-L-alanyl-D-glutamate–2,6-diaminopimelate ligase	E
6.3.2.8	murC*	UDP-N-acetylmuramate–L-alanine ligase	E
6.3.2.9	murD*	UDP-N-acetylmuramoylalanine–D-glutamate ligase	E

The EC (Enzyme Commission) numbers represent the classification of P. aeruginosa enzymes according to the Nomenclature Committee of the International Union of Biochemistry and Molecular Biology (IUBMB). Gene, product, and DrugBank Inhibitor Status were retrieved from UniProt and DrugBank databases, respectively. Abbreviations: experimental (E) and approved (A).

**Table 3** shows common targets with approved drugs. It is worth mentioning that several approved drugs have been identified; some of them are potential candidates for drug repositioning. Another relevant remark is the fact that most targets are also associated with experimental drugs.

**Table 3:** Putative targets with approved drugs common to all Tier 2 metabolic network models of *P. aeruginosa*. Targets marked with asterisks are also associated with drugs in the experimental stage. Drugs marked with double asterisks are most probably artifacts inherited from DrugBank.

EC number	Gene name	Approved drug
1.3.1.98	murB*	Flavin adenine dinucleotide**
1.5.1.3	folA*	Levoleucovorin
1.5.1.3	folA*	Isoniazid
2.3.1.41	fabB*	Cerulenin
2.5.1.15	*folP*	Sulfacytine
2.5.1.15	*folP*	Sulfaphenazole
2.5.1.15	*folP*	Sulfamethoxazole
2.5.1.15	*folP*	Sulfanilamide
2.5.1.15	*folP*	Sulfacetamide
2.5.1.15	*folP*	Sulfamethazine
2.5.1.15	*folP*	Sulfamethizole
2.5.1.15	*folP*	Sulfisoxazole
2.5.1.15	*folP*	Sulfamerazine
2.5.1.7	murA*	Fosfomycin
2.6.1.85	*pabB*	Formic acid**
4.2.1.24	hemB*	Formic acid**
4.2.3.5	*aroC*	Riboflavin monophosphate**
5.3.1.6	rpiA*	Citric acid**

The EC (enzyme commission) numbers represent the classification of P. aeruginosa enzymes according to the Nomenclature Committee of the International Union of Biochemistry and Molecular Biology (IUBMB).Gene and associated drug names were retrieved from UniProt and DrugBank databases, respectively.

Another noteworthy observation is that a considerable number of approved drugs in **Table 3** are most probably artifacts from the DrugBank database. For instance, flavin adenine dinucleotide (FAD), listed as an approved drug related to gene *murB*, is in fact approved for use in Japan under the trade name adeflavin as an ophthalmic treatment for vitamin B2 deficiency, it is just a cofactor for the product of gene *murB*, the enzyme UDP-N-acetylenolpyruvoylglucosamine reductase. All similar cases are highlighted with double asterisks in **Table 3**. This observation only reinforces a known limitation of all computational methods relying on databases at least partially annotated using automated workflows.

### Analysis of the Metabolic Network Model of the Multidrug-Resistant Strain *P. aeruginosa* CCBH4851

Considering the curated version of the metabolic network of multidrug-resistant strain *P. aeruginosa* CCBH4851, 17 unique targets were identified using the FBA+FVA method, while the FBA-only method returned 50 unique potential targets. Among those results, it is important to highlight four potential targets: *asd*, *ispE*, *fabA*, and *dapA*. Both *asd* and *dapA* are involved in the L-lysine biosynthesis *via* DAP pathway, which synthesizes L-lysine from aspartate and pyruvate. In bacteria, the lysine biosynthesis pathway yields the important metabolites meso-2,6-diaminopimelate (meso-DAP) and lysine. Lysine is utilized for protein synthesis in bacteria and forms part of the peptidoglycan cross-link structure in the cell wall of most gram-positive species, whilst meso-DAP is the peptidoglycan cross-linking moiety in the cell wall of gram-negative bacteria (Dogovski et al., 2012). This pathway is utilized by most bacteria, some archaea, some fungi, some algae, and plants (Liu et al., 2010b), and therefore are suitable candidates for therapeutic targets. Only experimental drugs are available to both targets.

*ispE* encodes a cytoplasmic kinase of the MEP pathway that is involved in the biosynthesis of the isoprenoids used by many gram-negative bacteria (including *P. aeruginosa*) (Heuston et al., 2012). Because isoprenoids are involved in a wide variety of vital biological functions, the seven enzymes without close human homologs that participate in their metabolism (encoded by *dxr, ispC, ispD, ispE, ispF, ispG, ispH* genes) are favorable candidate drug targets and several inhibitors have been already reported (Masini and Hirsch, 2014). Specifically for *ispE*, only experimental drugs are available.

*fabA* participates in fatty acid synthesis (FAS) processes, which includes also *fabB, fabD, fabI*, and *fabH*. The proteins encoded by these genes have an essential role during the synthesis of bacterial phospholipid membranes, lipopolysaccharide (LPS), and lipoproteins, thus representing attractive targets due to the structural differences between the human and bacterial proteins and the essentiality of FAS (Zhang et al., 2006; Leibundgut et al., 2008). Only experimental drugs are available to this target.

All four potential targets described above are reported to be overexpressed in *K. pneumoniae* when the pathogen is exposed to polymyxin B (Ramos et al., 2018), which is considered as a “last resort” antibiotic for infections caused by Carbapenem-resistant *Enterobacteriaceae*. Indeed, it has been shown that *P. aeruginosa* CCBH4851 is sensible only to polymyxin B (Silveira et al., 2014). This observation can be of interest in a combination therapy perspective when dealing with resistant *P. aeruginosa* infections, possibly acting synergistically with other drugs. An interesting observation is that the same target may be associated with similar reactions in both Tier 2 P*. aeruginosa* CCBH4851 and *K. pneumoniae*. For instance, *asd* is associated with the aspartate-semialdehyde dehydrogenase reaction in both metabolic networks, but reactants, products, and directionality differ. On the other hand, reactions associated with *fabA* differ in both metabolic network models. The gene *fabA* is associated to 13 reactions in *K. pneumoniae* and nine reactions in Tier 2 *P. aeruginosa* CCBH4851.

Another interesting observation is that the above targets have been identified by the FBA-only method. Only *dapA* is included in FBA+FVA results. One possible inference from this fact is that *dapA* should be prioritized over the other targets. Nevertheless, it also highlights the importance of considering both methods when looking for new potential targets.

A fifth target worth mentioning is *algC*, which encodes a highly reversible phosphoryltransferase. The phosphomannomutase activity produces a precursor for alginate polymerization; the alginate layer causes a mucoid phenotype and provides a protective barrier against host immune defenses and antibiotics. It is involved in core LPS biosynthesis due to its phosphoglucomutase activity and is essential for rhamnolipid production, an exoproduct correlated with pathogenicity (Olvera et al., 1999). It is also required for biofilm production (Davies and Geesey, 1995). This particular target was identified using the FBA-only method. Only experimental drugs are available to *algC*.

### Analysis of the Tier 3 *P. aeruginosa* CCBH4851 Metabolic Network

To evaluate the robustness of FindTargetsWEB regarding Tier 3 networks, which generally are networks generated automatically without manual curation, FindTargetsWEB processed a preliminary version of the metabolic network model of *P. aeruginosa* CCBH4851, which precedes the Tier 2 network described previously. This network is the only one in this paper which was processed using step 6b (algorithm 2) of the overall method. The growth rate of the Tier 3 version of the *P. aeruginosa* CCBH4851 network is 1.757 h^-1^, which is less consistent to the biology of *P. aeruginosa* spp. than the growth rate obtained by the Tier 2 version of the network. The processing of this Tier 3 network generated 32 targets in the FBA+FVA analysis, and 48 targets using the FBA-only method. It is remarkable that this less curated version of *P. aeruginosa* CCBH4851 network generated more potential targets in the FBA+FVA analysis than the corresponding Tier 2 network.

Among targets identified using the FVA+FBA method, 10 targets are common between the Tier 2 and Tier 3 networks. For the FBA-only analysis, 21 targets are common between the two versions. It is worth mentioning that many targets found in Tier 2 networks are present in the analysis of the CCBH4851 Tier 3 network, which corroborates the relevance of the targets found even in draft versions of metabolic networks. This comparison also highlights the importance of careful curation of automatically generated metabolic networks. For instance, from the targets discussed in the previous subsection, only *dapA* is present as a potential target in the Tier 3 network.

### Analysis of Metabolic Network Models of *K. pneumoniae* and *H. influenzae*

Metabolic networks of bacteria other than *P. aeruginosa* were also processed using FindTargetsWEB. In the previous subsections, results for *P. aeruginosa* metabolic network models were presented, but it is also possible to analyze networks of other species of bacteria. In this subsection, FindTargetsWEB results for a metabolic network reconstruction of *K. pneumoniae *MGH78578—iYL1228 (BioModels ID 1507180054) (Liao et al., 2011) and *H. influenzae*—iCS400 (BioModels ID 1507180053) (Schilling and Palsson, 2000) are presented (**Table 4**).

**Table 4:** List of EC numbers, product, and DrugBank inhibitor status for putative targets for metabolic network models of *K. pneumoniae *and *H. influenzae*. All targets listed in this table are included in the results of both FBA+FVA and FBA-only methods.

EC number	Gene name	Product	DrugBank inhibitor	Species
1.3.1.98	*murB*	UDP-N-acetylenolpyruvoylglucosamine reductase	A/E	*K. pneumoniae*
2.3.1.117	*dapD*	2,3,4,5-tetrahydropyridine-2,6-dicarboxylate N-succinyltransferase	E	*K. pneumoniae*
2.3.1.129	*lpxA*	Acyl-[acyl-carrier-protein]–UDP-N-acetylglucosamine O-acyltransferase	E	*K. pneumoniae*
2.3.1.179	*fabF*	3-oxoacyl-[acyl-carrier-protein] synthase 2	A/E	*K. pneumoniae*
2.3.1.41	*fabB*	3-oxoacyl-[acyl-carrier-protein] synthase 1	A/E	*K. pneumoniae*
2.7.2.8	*argB*	Acetylglutamate kinase	E	*K. pneumoniae*
2.7.4.9	*tmk*	Thymidylate kinase	E	*K. pneumoniae*
4.2.1.59	*fabA*	3-hydroxydecanoyl-[acyl-carrier-protein] dehydratase	E	*K. pneumoniae*
6.3.2.13	*murE*	UDP-N-acetylmuramoyl-L-alanyl-D-glutamate–2,6-diaminopimelate ligase	E	*K. pneumoniae*
6.3.2.8	*murC*	UDP-N-acetylmuramate–L-alanine ligase	E	*K. pneumoniae*
6.3.2.9	*murD*	UDP-N-acetylmuramoylalanine–D-glutamate ligase	E	*K. pneumoniae*
1.5.1.3	*folA*	Dihydrofolate reductase	A/E	*H. influenzae*
2.7.4.9	*tmk*	Thymidylate kinase	E	*H. influenzae*
2.7.7.38	*kdsB*	3-deoxy-manno-octulosonate cytidylyltransferase	E	*H. influenzae*
6.1.1.10	*metG*	Methionine–tRNA ligase	E	*H. influenzae*
6.1.1.2	*trpS*	Tryptophan–tRNA ligase	A/E	*H. influenzae*
6.1.1.21	*hisS*	Histidine–tRNA ligase	E	*H. influenzae*
6.1.1.3	*thrS*	Threonine–tRNA ligase	E	*H. influenzae*
6.3.5.2	*guaA*	GMP synthase [glutamine-hydrolyzing]	A	*H. influenzae*

The EC (Enzyme Commission) numbers represent the classification of K. pneumoniae and H. influenzae enzymes according to the Nomenclature Committee of the International Union of Biochemistry and Molecular Biology (IUBMB). Gene, product, and DrugBank Inhibitor Status were retrieved from UniProt and DrugBank databases, respectively. Abbreviations: experimental (E) and approved (A).

For *K. pneumoniae*, a total of 45 unique potential targets were found using the FBA+FVA method and also 45 for the FBA-only method. Some of the more representative targets are listed in **Table 4** (complete results are available as **Supplementary Material**).

Several targets identified in **Table 4** are worth mentioning. For instance, the cytoplasmic enzyme encoded by *lpxA* gene is involved in the initial steps of lipid A production through the Raetz pathway. As stated in the previous subsection, *fabA*, *fabB*, and *fabF* participate in FAS processes and represent attractive targets due to the structural differences between the human and bacterial proteins and the essentiality of FAS. The cytoplasmic protein N-acetylglutamate (NAG) kinase (encoded by *argB*), which promotes phosphorylation of NAG in a rate-limiting step of bacterial L-arginine production, occurs through acetylated intermediates, unlike mammals which use non-acetylated intermediates, and for this reason, it was previously considered a candidate drug target (Marcos et al., 2010). Indeed, Ramos et al. (2018) identified several potential targets found by FindTargetsWEB as priority targets for *K. pneumoniae*. Examples are *dapD, lpxA, fabA, fabB, tmk, murE*, and *murD*. Their analysis included a reconstruction of the metabolic network model of *K. pneumoniae* Kp13 and an essentiality analysis based on literature search. A target prioritization pipeline was proposed that takes into account gene essentiality, topological measures, literature information, and gene expression data. It is worth noting that neither FBA nor FVA were used in their analysis.

For the metabolic network model of *H. influenzae*, 16 unique potential targets were found by FindTargetsWEB for both FBA+FVA and FBA-only methods (**Table 4**). Complete results are available as **Supplementary Material**.

It is worth mentioning that the genes *folA, tmk, kdsB, metG, thrS*, and *guaA* were identified as essential for *H. influenzae* growth and survival by Akerley and colleagues (2002), using a high-density transposon mutagenesis strategy. Another relevant observation is the presence of potential targets common to *K. pneumoniae* (*tmk*) and *P. aeruginosa* (*folA*). Both methods, FBA+FVA and FBA-only, generate exactly the same results. Therefore, the FVA ranges for all targets in **Table 4** are equal to zero.

### Analysis of a Host-Pathogen Integrated Metabolic Network Model

FindTargetsWEB is also capable of processing integrated metabolic network models. The analysis presented in this subsection used a host-pathogen genome-scale reconstruction, iAB-AMØ-1410-Mt-661 (BIOMODELS ID 1011090001), which integrates a cell-specific alveolar macrophage model, iAB-AMØ-1410, from the global human metabolic reconstruction, with an *M. tuberculosis* H37Rv model, iNJ661 (Bordbar et al., 2010). The integrated host-pathogen network enables simulation of the metabolic changes during infection.

A total of 35 unique potential targets was identified by FindTargetsWEB on the integrated model by both the FBA+FVA and FBA-only methods (complete results are available as **Supplementary Material**). Several potential targets found by FindTargetsWEB in the host-pathogen integrated model have been previously reported in the literature as essential to *M. tuberculosis* survival (Bordbar et al., 2010; Sassetti and Rubin, 2003). Examples are *nrdE*, *mmaA2*, *mmaA3, aroQ*, and *ahcY*, from which only *mmaA2* and *mmaA3* have approved drugs. It is worth highlighting that the selection of potential targets of FindTargetsWEB depends not only on network analysis, but also on data retrieved from DrugBank and additional filters, such as a low level of similarity with human proteins.

### Analysis of the Metabolic Network Model of a Gram-Positive Bacterium

None of the results presented in the previous subsections include gram-positive bacteria. To emphasize FindTargetsWEB flexibility, in this subsection, we present results from the metabolic network model analysis of a gram-positive pathogen. *S. aureus* is a pathogenic gram-positive bacterium that causes a variety of disease conditions both in hospital settings and in the community at large. The metabolic model iSB619 (BIOMODELS ID 1507180070) (Becker and Palsson, 2005), reconstructed from the strain N315, was processed using FindTargetsWEB. Complete results for both FBA-only and FVA+FBA are available as **Supplementary Material**.

A total of 27 unique potential targets were generated using the FBA-only method. The FBA+FVA analysis returned 22 unique targets. Some potential targets are common to gram-negative bacteria (such as *murB*, *aroC*), while others such as *mvaA* (locus tag SA2333 for the N315 strain, SAOUHSC_02859 for the NCTC8325 strain), *tkt* (SA1177, SAOUHSC_01337), and *dfrA* (SA1259, SAOUHSC_01434) are defined as essential for *S. aureus* in both minimal and rich medias (Becker and Palsson, 2005). Regarding the metabolic network models analyzed in this manuscript, the potential targets *mvaA*, *tkt*, and *dfrA* only appear in the *S. aureus* metabolic network model.

### Analysis of the Metabolic Network Model of a Non-Pathogenic Bacteria

The pseudomonads include a diverse set of bacteria whose metabolic versatility and genetic plasticity have enabled their survival in a broad range of environments. Many members of this family are able to either degrade toxic compounds or to efficiently produce high value compounds and are therefore of interest for both bioremediation and bulk chemical production. *P. putida* is a representative of those industrially relevant pseudomonads. In this subsection, an analysis of the metabolic network model of the *P. putida* KT2440 (Puchałka et al., 2008), named iJP815 (BIOMODELS ID 1507180044), is compared to the previous analysis of a pathogenic member of the family, *P. aeruginosa*. Complete results for the analysis of the *P. putida* metabolic network model is available as supplementary material.

A first comparison between *P. putida* e *P. aeruginosa* metabolic network models is the number of potential targets. The analysis of the metabolic network model of *P. putida* returned a comparable number of potential targets: 52 for FBA-only, 50 for the FBA+FVA method (see **Table 1**). Indeed, the size of the metabolic network model iJP815 is comparable with other *P. aeruginosa *metabolic networks: 824 intracellular and 62 extracellular metabolites connected by 877 reactions. Other interesting observation is that some targets present in the multidrug-resistant *P. aeruginosa* CCBH4851 are absent in *P. putida*, despite the comparable number of potential targets. Remarkable examples are *asd*, *ispE*, *fabA*, *dapA*, and *algC*. Indeed, from the 25 targets common to all Tier 2 *P. aeruginosa* metabolic network model displayed in **Table 2** (FBA-only method), only 18 are also potential targets for the *P. putida* KT2440 metabolic network model.

## Discussion

Several advantages of the proposed method can be highlighted: first the robustness of the system, which can identify potential targets even for draft (Tier 3) networks, pointing out that such metabolic network models are very common and are the only models available for some organisms. The system is deployed as a web application and is asynchronous: the user is notified when results are available. The performance of the system is optimized, since the COBRApy framework can make use of multiple cores available in the host machine, and it is able to process the metabolic network of various bacteria, as described in the previous section. The only requirement is the availability of an SBML level 3 file describing the corresponding genome-scale metabolic network. The user interface is straightforward (see **Figures 1** and **2**), and the user should only provide a name, an e-mail address, and the corresponding SBML file. The user should also indicate the species of bacterium associated with the metabolic network model. FindTargetsWEB is a highly flexible tool, capable of processing genome-scale metabolic network models of gram-negative bacteria, gram-positive bacteria, bacteria not classified as either gram-positive or gram-negative, and even integrated host-pathogen genome-scale metabolic network models.

Other proposals for the analysis of metabolic networks at genomic scale are available in the literature. Chavali et al. (2012) used FBA and FVA for identification of potential targets, but their application does not propose any drugs for the targets found neither describes the potential targets in detail. The procedure reported in (Oberhardt et al., 2010) describes a processing similar to the one proposed in this work up to the EC number mapping step, and then uses graphical tools to identify the potential targets for *E. coli* and *Bacillus subtilis*, without pinpointing any potential drug. Ramos et al. (2018) propose a method to identify drug targets in metabolic network model of *K. pneumoniae*. However, their method is not automated, and it was not applied to other species of bacteria. None of these works go as far as FindTargetsWEB, which can process metabolic network models of several species of bacteria, identify potential targets, confirm homology with the analyzed gene, and identify all available drugs for the potential target in a fully automated manner.

Regarding the options to identify potential targets, i.e., *FBA+FVA* and *FBA-only*, one can conclude that the FBA+FVA method represents a way to prioritize the targets identified by the FBA-only method, since the set of targets identified by FBA+FVA is a proper subset of the set of targets identified by FBA-only. However, as stated in the detailed description of the targets of the Results section, potential targets that are associated with the FBA-only method and do not appear as results of the FBA+FVA method should not be disconsidered. Many important targets described in the literature have a FVA range greater than zero, and a careful analysis of both sets of potential targets is advised.

Several of the approved drugs identified by FindTargetsWEB are already used against *P. aeruginosa* and other bacteria and can be effective against non multidrug-resistant strains. As expected, for the multidrug-resistant strain, most of the approved drugs are not effective. For instance, it is known that *P. aeruginosa* can be resistant to both to trimethoprim and sulfamethoxazole (see **Table 3**) due to the MexAB-OprM multidrug efflux system (Köhler et al., 1996). Nevertheless, FindTargetsWEB also pinpoints a large number of experimental drugs that can be effective. Actually, most of the targets identified by FindTargetsWEB for all strains are associated to experimental drugs and may represent new therapeutic options. Clearly, additional *in vitro* and *in vivo* testings are needed in order to confirm the experimental drugs as new therapeutic options.

Additional information provided by FindTargetsWEB can also be considered in the definition of new strategies to fight multidrug-resistant bacteria. Information such as pathway, target function, and catalytic activity can be considered in order to devise a multi-target strategy, which can be very effective in some scenarios. As an example of a multi-target strategy, in bacteremia caused by *P. aeruginosa*, the combination of efflux pump inhibitors and iron chelators has been proposed to control the infection process in view of the overexpression of the MexAB-OprM efflux system during iron deprivation (Liu et al., 2010a). Indeed, several targets in the analysis of results for *P. aeruginosa* are related to different cellular functions. Targeting several cellular functions and processes at the same time can be a more promising strategy than considering only one isolated target. For instance, it is known that inhibiting bacterial growth can accelerate the process of biofilm formation (Xu et al., 2013). Therefore, the pathogen can form a biofilm before it is eliminated. Multi-target therapies are already commonplace in treating bacteria infections, and the wealth of information provided by FindTargetsWEB can be used to define new multi-target treatments not considered before. For instance, *algC* (*P. aeruginosa* CCBH4851, PA14, and PAO1-2017 metabolic networks) is both essential to metabolic growth and biofilm formation, according to the FUNCTION field returned by FindTargetsWEB and literature sources (Davies and Geesey, 1995). Therefore, a targeting strategy based on other genes may consider also targeting *algC* to prevent biofilm formation.

## Concluding Remarks

FindTargetsWEB is a user-friendly web application that combines bioinformatics and systems biology, providing insights of new therapeutic targets for multidrug-resistant bacteria, increasing the available therapeutic options. By identifying more effectively potential targets along with candidate active compounds for posterior experimental confirmation, this tool prevents exhaustive bacterial drug screening. Importantly, FindTargetWEB can also be applied to the study of other bacteria due to the flexibility proposed by computational modeling, serving as a base for other relevant studies. In addition, it will serve as a starting point for the creation of even more complete applications in a web environment, such as one capable of processing integrated computational models and retrieving data from more databases.

## Availability and Requirements

Project name: FindTargetsWEB

Project home page: http://pseudomonas.procc.fiocruz.br/FindTargetsWEB

Operating system: e.g. Web-based, Platform independent

Programming language: Python 3.6

Other requirements: An updated web browser (e.g. Google Chrome, Mozilla Firefox, Apple Safari, Microsoft Edge)

License: Not Applicable

Any restriction to use by non-academics: Not Applicable

The user must provide a SBML level 3 file describing the metabolic network reconstruction and an e-mail address to which the results will be forwarded.

## Data Availability Statement

All datasets generated for this study are included in the manuscript and the supplementary files.

## Author Contributions

TCM, FPSJ, and FABS designed the system. TCM was the main programmer. MWC and ADC-A tested the system and evaluated its correctness. All authors have equally participated in the writing of this paper.

## Funding

The authors would like to thank CAPES, FAPERJ, CNPq and FIOCRUZ (INOVA-FIOCRUZ VPPCB-007-FIO-18-2-29) for financial support.

## Conflict of Interest Statement

The authors declare that the research was conducted in the absence of any commercial or financial relationships that could be construed as a potential conflict of interest.
